# Trends in penile cancer: a comparative study between Australia, England and Wales, and the US

**DOI:** 10.1186/s40064-015-1191-4

**Published:** 2015-08-14

**Authors:** James Sewell, Weranja Ranasinghe, Daswin De Silva, Ben Ayres, Tamra Ranasinghe, Luke Hounsome, Julia Verne, Raj Persad

**Affiliations:** Department of Surgery, Western Hospital, Gordon Street, Footscray, VIC 3011 Australia; Monash Health, Melbourne, Australia; La Trobe Business School, La Trobe University, Melbourne, Australia; St George’s Hospital, London, UK; Department of Neurology, West Virginia University, Morgantown, USA; South West Knowledge and Intelligence Team, Public Health England, Bristol, UK; University Hospital Bristol, Bristol, UK

**Keywords:** Penile neoplasms, Circumcision, Male, Carcinoma, Squamous cell, Male, Incidence

## Abstract

**Purpose:**

To investigate and compare the trends in incidence and mortality of penile cancer between Australia, England and Wales, and the US, and provide hypotheses for these trends.

**Methods:**

Cancer registry data from 1982 to 2005 inclusive were obtained from Australia, England and Wales, and the United States. From these data, age-specific, -standardised and mortality:incidence ratios were calculated, and compared.

**Results:**

The overall incidence of penile cancer in England and Wales (1.44 per 100,000 man-years) was higher than in Australia (0.80 per 100,000), and the US (0.66 per 100,000). Incidence of penile cancer in all three countries has remained relatively stable over time. Similarly, although the mortality rates were also higher in England and Wales (0.37 per 100,000 man-years) compared to Australia (0.18 per 100,000) and the US (0.15 per 100,000), the mortality/incidence ratios were similar for all three countries.

**Conclusions:**

Penile cancer incidence is low, affecting mainly older men. Rates differ between the three countries, being twice as common in England and Wales as in the other studied regions. Circumcision rates have a potential influence on these rates but are not the sole explanation for the variation.

## Background

Invasive squamous cell carcinoma of the penis (hereafter referred to as penile cancer) is a rare disease, the reported rates of which vary widely between countries (Parkin and Muir 1992). Penile cancer predominantly affects older men, but is a disease that carries with it a high psychological impact of treatment. Although penile preserving surgery is becoming increasingly common, partial or total penectomy is sometimes required, in addition to potential chemoradiotherapy for patients with advanced nodal disease.

Multiple causative factors for penile cancer have been identified, mostly relating to inflammation of the penis, although cigarette smoking and low socioeconomic status have also been linked to the disease (Dillner et al. 2000; Tsen et al. 2001; Madsen et al. 2008; Maden et al. 1993; Aynaud et al. 1999; Narayana et al. 1982; Daling et al. 2005). In particular, the association between phimosis and penile cancer has been well studied, and a causal link between the two conditions is widely accepted (Larke et al. 2011; Pizzocaro et al. 2010). A link between infant circumcision and penile cancer has been drawn, with a degree of controversy in the literature as some advocate infant circumcision as preventative of penile cancer (Dodge et al. 1963; Morris et al. 2012; Svoboda and Van Howe 2013).

Here we compare the incidence and mortality of penile cancer over a number of years, between Australia, the US and England and Wales, three Western countries with broadly similar populations. We also discuss causative factors for the disease and look in further depth at circumcision, comparing rates of circumcision between the three countries to identify if there is an impact on the incidence of penile cancer.

## Methods

Data regarding penile cancer diagnoses and mortality from 1982 to 2005 were obtained from the Australian Institute of Health and Welfare National Mortality Database, the England and Wales Cancer Registry, and from the Surveillance, Epidemiology, and End Results (SEER) Registries in the US. The penile cancer diagnoses for the UK were available only from 1995 to 2003, however mortality data was available from 1982–2005. Nine registries were used when analysing the SEER database (Atlanta, Connecticut, Detroit, Hawaii, Iowa, New Mexico, San Francisco-Oakland, Seattle-Puget Sound, and Utah).

The Australian Institute of Health and Welfare (AIHW) database is the central cancer registry for all cancer incidence and deaths and collates data by all state-based cancer registries in Australia and is a validated and reputable source of data (Australian Institute of Health and Welfare 2012). Similarly, the England and Wales Cancer Registry and SEER database are also previously validated and reputable cancer registry databases in England (Office of National Statistics 2014) and the US (National Cancer Institute 2010), respectively.

### Statistics

Age-specific rates were calculated using 5-year age groups capped with an 85 + group and age-standardised incidences were calculated using the Segi World Standard Population (Segi et al. 1957). For the incidence trends, crude incidence was calculated, as age-specific incidence was not available for each year covered.

Mortality rates were calculated per 100,000 man-years and mortality/incidence ratios were calculated using crude incidence and mortality rates.

Incidence rates were calculated for 1- and 5-year periods and incidence trends were calculated using linear regression. Statistics were calculated using one-way ANOVA. Standard error is displayed on figures where applicable.

Statistics were calculated using Microsoft Excel, version 14.1 for Mac OSX (2011).

### Literature review

To investigate the prevalence of circumcision, a literature search was performed using the pubmed database with the MeSH terms “circumcision” and “prevalence” AND/OR “incidence”. Papers relevant to the 3 countries being studied were selected and reviewed. Twenty-three articles were reviewed. To ensure relevant papers were not missed, the references of the papers were also reviewed. Ten articles met the search criteria. Non-English articles were excluded.

## Results

**Fig. 1:**
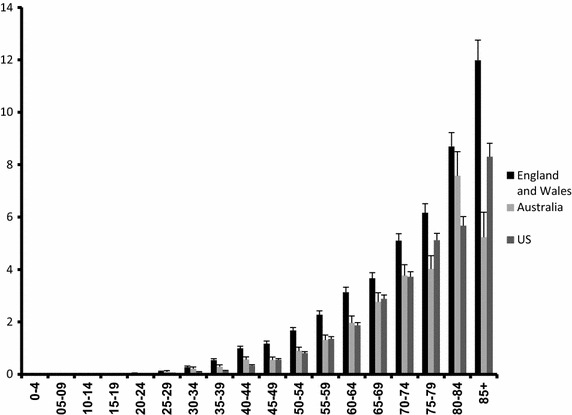
Age-specific incidence of penile cancer (1996–2003) per 100,000 man-years.

The incidence of penile cancer increases with age. (Fig. 1) In England and Wales and the US, the peak incidence is in the 85+ age group, while in Australia the incidence peaks in the 80–84 age group.

**Fig. 2:**
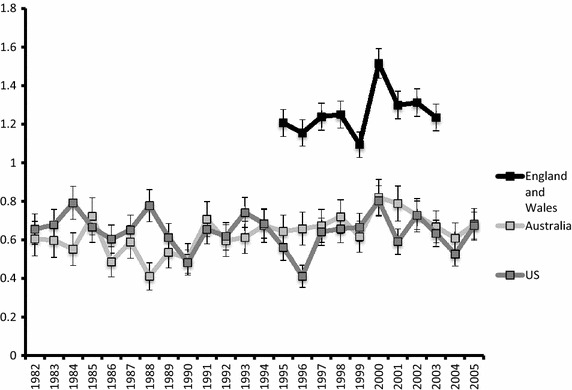
Incidence of penile cancer in three countries by year, per 100,000 man-years.

Data on incidence of penile cancer were available between 1982 and 2005 in Australia and the US, but only for 1995 to 2003 in England and Wales (Fig. 2). The incidence in England and Wales was significantly higher than the incidence in Australia and the US (p < 0.001). Age-standardised incidence was 0.54 in Australia, 0.52 in the US, and 0.87 in England and Wales (Table 1).

**Table 1:** 5-year incidence, mortality, and mortality/incidence ratios and incidence trends

	5-year incidence			Mortality rates			M/I ratio			Incidence trend		
Year	AUS	US	E&W	AUS	US	E&W	AUS	US	E&W	AUS	US	E&W
1984–88	0.55	0.70	–	0.19	0.16	0.42	0.36	0.32	–	–	–	–
1989–93	0.59	0.62	–	0.20	0.16	0.40	0.33	0.33	–	7.0%	−10.9%	–
1994–98	0.67	0.59	1.21	0.16	0.15	0.35	0.23	0.35	0.25	14.0%	−4.9%	–
1999–2003	0.72	0.69	1.29	0.17	0.15	0.35	0.24	0.30	0.24	7.4%	15.7%	6.4%
2004–05	0.65	0.58	–	0.13	0.15	0.30	0.20	0.34	–	−10.9%	−15.2%	–
ANOVA	p < 0.001			p < 0.001			p = 0.02			Trend by linear regression (p-value)		
Crude incidence	0.80	0.66	1.44									
Overall age-standardised rate (S.E)	0.54 (0.15)	0.52 (0.06)	0.87 (0.11)							0.007 (p = 0.006)	−0.002 (p = 0.52)	0.016 (p = 0.32)

Incidence data only available for England and Wales from 1995–2003.

**Fig. 3:**
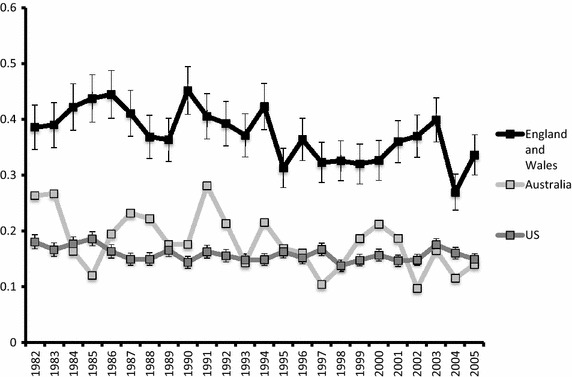
Mortality rates in 3 countries by year, per 100,000 man-years. Note: standard error not displayed on Australian data as cases per year not available.

Mortality rates were available between 1982 and 2005 in all studied regions (Fig. 3). Mortality is higher in England and Wales than in Australia or the US (p < 0.001). Mortality/incidence (MI) rates were similar in all studied regions (p = 0.02) (Table 1).

Incidence trends were calculated using linear regression, and proved stable in England and Wales and in the US. A very small but statistically significant increase was observed in the Australian data (0.7% per year, p = 0.006) (Table 1).

## Discussion

The data we present here clearly show a significant difference between the incidence of penile cancer in the US, England and Wales, and Australia. While the US and Australian incidences are similar, the incidence in England and Wales is considerably higher. The rates in England and Wales correlate with other published European data; rates in Denmark are similarly high (1.3 per 100,000 man-years) (Baldur-Felskov et al. 2012). Of note, the MI indices of all three countries are similar, suggesting that the treatment and stage of diagnosis of the cancers in all three countries are similar. The incidences of penile cancer in all three studied areas are much lower than the published rates from Africa. The peak in age-specific incidence varied between Australia, the US and England and Wales, peaking in the 80–84 age group in Australia and the 85+ age group in the US and England and Wales.

Trends in incidence show that the rates of penile cancer were stable over the studied period in England and Wales and the US. The Australian data show a very small but statistically significant increase over time. The fact that there has been little change in penile cancer incidence over time suggests that either the aetiological factors are unchanging or that despite changes in individual factors, the overall balance of protective and causative factors has been maintained.

The Australian and British populations are broadly similar in their rates of many cancers, with the notable exception of sun-related skin cancers (Parkin 2001; Society AC 2011). The ethnic mix within the population of the two countries, the diet, and the lifestyles are also similar, meaning that genetic or broad lifestyle factors are less to account for this difference in incidence (Dwyer and Hetzel 1980). Various aetiological factors are proposed to play a role in penile cancer. These generally relate to inflammatory processes that occur in the penis, and include HPV infection, phimosis, lichen sclerosus, and poor penile hygiene (Dillner et al. 2000; Tsen et al. 2001; Madsen et al. 2008; Maden et al. 1993; Aynaud et al. 1999; Narayana et al. 1982; Daling et al. 2005). It seems unlikely that there is a significant and persistent difference in penile hygiene between England and Wales and Australia, so this is unlikely to account for the difference in incidence.

Smoking rates have been declining since at least the 1980s, in all three regions (Ng et al. 2014). The rates are similar in each region, meaning that difference in smoking rates is unlikely to account for a difference in penile cancer rates (West et al. 2007; Jha et al. 2002).

Little data has been published about incidence trends for HPV infection, especially in males. A study from Finland, where large serum banks have been collected for many years, suggest increasing rates of HPV infection between the 1980s and 1990s, in women of childbearing age (Laukkanen et al. 2003). Due to widely varying prevalence of HPV infection around the world, this may not hold true in the countries studied in this paper (Clifford et al. 2005). With HPV vaccination programmes becoming widespread, it is likely that these rates are falling. Nevertheless, given the long lag time between HPV infection and penile cancer developing, no effect is likely to be seen for many years to come, and this is therefore unlikely to have affected the results of this study.

A link has been drawn between a lack of neonatal/infant circumcision and an increase incidence of penile cancer. It has been suggested that infant circumcision rather than adult circumcision reduces the risk of penile cancer (Maden et al. 1993; Larke et al. 2011; Schoen et al. 2000). A meta-analysis by Larke et al. (2011) shows an odds ratio of 0.33 for penile cancer in patients who underwent infant circumcision. Much of the early literature concerning the link between penile cancer and circumcision has come from studies conducted in the US and Africa, where different cultural groups routinely perform infant circumcision, and are noted to have lower incidence of penile cancer (Dodge et al. 1963; Wolbarst 1932). However, the findings from Africa may not be directly applicable to a Western setting, given that recorded incidences of penile cancer in African countries are much higher than the global average (Wabinga et al. 2000; Parkin et al. 2008). Despite this, various organisations have published position papers and recommendations regarding infant circumcision, often mentioning an association with penile cancer, but the topic remains under debate (WHO/UNAIDS technical consultation 2007; Fetus and Newborn Committee, Canadian Paediatric Society 1996; American Academy of Pediatrics 1999; Lannon et al. 2000).

To investigate whether circumcision rates could be having an impact on our data, we conducted a review of the available literature regarding circumcision rates. Limited data have been published regarding circumcision rates, but those data which were available is summarised in Table 2. The data available were a mix of population prevalence and yearly incidence, with many of the incidence data being in-hospital neonatal/infant circumcision rates. As the vast majority of circumcisions occur in infancy, incidence rates should roughly correlate with prevalence of circumcision (i.e.: if 10% of the male population are circumcised in infancy, then it would be expected that 10% of the population at a given point in time would be circumcised). Some prevalence data come from cross-sectional studies or surveys, and other data represent expert estimation.

**Table 2:** Circumcision rates in Australia, the UK, and the US

Country	Author	Year	Circumcision prevalence/incidence
Australia	Richters et al. (2006)	2002	59% (prevalence in 16–19 year olds only 31%)
Australia	Spilsbury et al. (2003)	1994–1999	8–10% infant circumcision
Australia	RACP, Division of Infant and Child Health (2010)	2010	10–20% infant circumcision (estimated)
Australia	Darby (2011)	2000–2010	12% neonatal circumcision
UK	Gairdner (1949)	1949	20% (estimated)
UK	Dave et al. (2003)	2000	15.8% (prevalence in 16–19 year olds 11.7%)
UK	Rickwood et al. (2000)	2000	3.8% (circumcision before 15)
UK	Cathcart et al. (2006)	1997–2003	3.9%, with 3.1% circumcision before 15
US	American Academy of Paediatrics (2012)	1998–2008	57% infant circumcision
US	American Academy of Paediatrics (2012)	1999–2004	79% prevalence
US	Centers for Disease Control (2011)	1999–2010	55.8–59.1% infant circumcision

The US and Australia had the highest prevalence and incidence rates, with prevalence in Australia 30–50%, and incidence of infant circumcision 10–20%. Prevalence in the US was 79% and incidence of infant circumcision 55–60%. The UK had much lower circumcision rates than the other two countries, with 15–20% prevalence and an incidence of just 3–4% infant circumcision. Data on England and Wales alone, separating out the rest of the UK, were not available. The fact that incidence in all cases was lower than prevalence is likely due to two factors: (1) reporting of only a subset of circumcisions (infant in-hospital), and discounting religious/cultural circumcisions that occur outside hospital, as well as adult circumcisions, and, (2) falling rates of circumcision. Infant and adult circumcision typically occur for different reasons. There is good inter-study correlation for each of the three countries. However, the studies cannot be aggregated in their results as different methods have been used to obtain values for incidence and prevalence.

As has been previously noted, infant circumcision has been posited to have a protective role in penile cancer (Maden et al. 1993; Larke et al. 2011; Pizzocaro et al. 2010). The rates of infant circumcision are widely different between Australia and the UK, which have broadly similar populations. The differing incidence of penile cancer between the two populations may therefore reflect the protective effect of neonatal circumcision in Australia.

The circumcision rates in the US are much higher than the rates in the other two countries. Although the population demographics and rates of other cancers in the US differ from the other two countries, the incidence of penile cancer is similar to that in Australia (Parkin and Muir 1992; Parkin 2001). It might be expected that if a strong protective effect of infant circumcision were present, incidence of penile cancer in the US would be considerably lower than in the other countries. However, this lack of difference is difficult to interpret, given the many demographic differences between the US population and the other two studied countries. A recent paper using in-depth analysis of the SEER nine registries between 1973 and 2002 found that the penile cancer rate was far higher in Hispanic whites than in other population groups within the US (Barnholtz-Sloan et al. 2007). This is a group that is not highly represented in either England and Wales or Australia, and this may obscure the interaction between circumcision rates and penile cancer incidence when the three regions are compared. It would be interesting to examine circumcision rates in this population for comparison with other populations in the US. However, these data were not available for this study.

Although we see only a tiny rise in penile cancer rates in Australia, and no statistically significant change in incidence in England and Wales as the rate of circumcision has fallen, it is entirely possible that this is due to the long lag between infant circumcision and the peak incidence of penile cancer. Over time, mortality from penile cancer has marginally reduced, presumably with improved treatment, possibly in assessing for and managing early nodal disease. With a generally noted decline in circumcision rates beginning in 1950 and continuing until the present, were a distinct correlation in penile cancer incidence to emerge, it would be likely to begin to be observed in 2020 and trend upwards until 2060.

Limitations from this study include the fact that the use of cancer registry databases, which, while widely accepted, relies on reporting of cancer, either when diagnosed or on a death certificate. This may result in under-reporting of disease rates.

## Conclusion

Penile cancer is a rare disease, the burden of which falls predominantly on older males. The rates have remained fairly stable between 1982 and 2005 in all three regions. In addition, mortality/incidence ratios were similar across all three regions, suggesting similar treatment outcomes.

England and Wales had the highest incidence and mortality rates of penile cancer compared to Australia and US and also the lowest infant circumcision rates and overall circumcision prevalence. This offers some support to previous data suggesting infant circumcision may reduce risk of penile cancer. However, despite having higher circumcision rates in the US, the incidence of penile cancer in Australia and the US were similar, suggesting that the penile cancer incidence is not solely attributable to circumcision rates.

It will be interesting to observe the trends in the incidence of penile cancer over the years to come, as the time interval between change in infant circumcision rates and development of penile cancer grows.
